# Anterior Segment Optical Coherence Tomography for the Quantitative Evaluation of the Anterior Segment Following Boston Keratoprosthesis

**DOI:** 10.1371/journal.pone.0070673

**Published:** 2013-08-05

**Authors:** Joann J. Kang, Norma Allemann, Thasarat Vajaranant, Jose de la Cruz, Maria Soledad Cortina

**Affiliations:** 1 Department of Ophthalmology and Visual Sciences, University of Illinois at Chicago, Chicago, Illinois, United States of America; 2 Department of Ophthalmology, Federal University of São Paulo, São Paulo, Brazil; Duke University, United States of America

## Abstract

**Objective:**

To quantitatively evaluate the anterior segment using anterior segment optical coherence tomography (AS-OCT) following Boston keratoprosthesis type 1.

**Methods:**

A retrospective study consisted of AS-OCT imaging at a single time point postoperatively in 52 eyes. Main outcomes measures include anatomical and functional anterior chamber depth (ACD), angle (ACA) and peripheral and proximal synechiae.

**Results:**

The mean time point of imaging was 19.3 months postoperatively. Average anatomical and functional ACD was 2.0 and 0.21 mm respectively, and mean ACA ranged from 2.5° to 6.14° in representative meridians. An average of 8.7 clock hours of angle closure was observed in the 25 eyes in which all meridians were imaged. The majority of eyes showed peripheral (86.5%) and proximal (67.3%) synechiae.

**Conclusions:**

AS-OCT is a useful tool for quantitative evaluation of anterior segment and angle after keratoprosthesis, which is otherwise poorly visible. The majority of eyes showed shallow ACD, extensive angle closure and synechiae formation.

## Introduction

The Boston keratoprosthesis (KPro) type 1 is an increasingly well-accepted surgical intervention for patients with severe corneal and ocular surface disease who are poor candidates for traditional penetrating keratoplasty (PKP). The KPro device consists of a separate front part and back plate (made of polymethyl methacrylate or titanium) that sandwiches a donor carrier corneal graft. [1] The central embedded stem provides an optically clear visual axis. The assembled KPro device is then sutured into place in the same method as a standard corneal transplant. Modifications made to the device design and improvements in postoperative management have led to better retention rates and reduced serious complications, leading to an increased number of KPro procedures performed in the United States and worldwide. [2].

However, visualization of the implanted Boston KPro, anterior segment and angle using conventional clinical examination techniques is limited; consequently, the interaction of KPro with surrounding anterior segment structures is not well understood. Anterior segment imaging including ultrasound biomicroscopy (UBM) and anterior segment optical coherence tomography (AS-OCT) has improved the evaluation of otherwise poorly visualized anterior chamber structures behind opacified corneas and implanted KPros. [3], [4] UBM uses 35–50 MHz of high-frequency ultrasound waves to obtain images that can identify the anatomical characteristics of the anterior chamber and angle. [5] It has been used successfully for the preoperative evaluation of candidates for KPro. [6] However, this technique is considered a contact technique because it requires immersion (water bath) or contact of the eye with a plastic balloon and needs a highly trained examiner. [7] Furthermore, imaging of the implanted KPro by UBM is limited because the prosthesis material causes artifacts impairing the visualization of anterior segment anatomical details. In comparison, AS-OCT allows noninvasive, noncontact, high-resolution cross-sectional imaging of the anterior segment. [8] Infrared (1310 nm) radiation is used to provide real-time images and allows for qualitative and quantitative assessments of the anterior chamber and angle structures.

Garcia et al. [3] compared these imaging modalities in two eyes with implanted KPro and found that AS-OCT was superior to UBM. They demonstrated that AS-OCT exhibited high-resolution images of the KPro device in vivo, including visualization of the anterior chamber, iris and angle, whereas UBM generated a poorer quality image of the implanted KPro device. Subsequently a few published case series have reported on AS-OCT imaging and Boston KPro, but the majority have focused on the donor-device interface. [9],[10],[11],[12] Recently, Panarelli et al. qualitatively evaluated the angle status at the horizontal meridian in a small case series. [13] However, to our knowledge, no previously published studies have used AS-OCT to quantitatively evaluate the anterior segment and angle following KPro implantation. Thus, the purpose of our study is to use AS-OCT to quantitatively evaluate iris characteristics and angle following implantation of Boston KPro in a larger case series.

## Methods

### Ethics Statement

Approval from the Institutional Review Board at the University of Illinois at Chicago was obtained. Exemption and waiver for HIPAA authorization and written consent were granted as retrospective collection of existing data met regulatory requirements. All clinical investigation was conducted according to the principles expressed in the Declaration of Helsinki.

A retrospective case series was conducted on 52 eyes of 49 patients who had implantation of primary (no prior history of a penetrating corneal procedure) or secondary Boston type 1 keratoprosthesis by two surgeons (MSC and JDC) at a single tertiary care institution between 2007–2011. Patients under the age of 18 years were excluded from the study. Standard surgical technique was used for all patients with a preference to implant 7 mm polymethylmethacrylate (PMMA) backplates, although 8.5 mm diameter PMMA backplates were also used.

All patients underwent AS-OCT imaging postoperatively at a single time point (Visante Omni, Carl Zeiss Meditec, Germany). Details of AS-OCT technology have been described previously. [8], [14] All images were acquired in standard light conditions using the anterior segment single, dual and/or quad scan protocol by two examiners (MSC, NA), usually without removal of the bandage contact lens. All eyes were scanned in the horizontal meridian (0° and 180°). Some eyes had additional scans in the vertical meridian (90° and 270°) and the oblique meridians (135° and 315°, 45° and 225°).

The cross sectional AS-OCT images with the best quality and/or greatest number of meridians scanned at a single time point were further analyzed using software provided by the manufacturer. This included evaluation of the anterior chamber angle (ACA) at the above specified meridians. In addition, the total number of clock hours of open, narrow or closed angle was recorded. A closed ACA on AS-OCT was defined as contact between the peripheral iris and angle wall anterior to the scleral spur (<1°). We defined an open angle as greater than 10° and a narrow angle between 1° and equal to or less than 10° on AS-OCT measurements. The anatomical anterior chamber depth (ACD; distance between the back of donor graft to the anterior iris plane) adjacent to the KPro front plate periphery and the functional ACD (distance between the posterior edge of the back plate and the anterior iris plane) were measured. The presence of proximal synechiae to the back plate (iris-back plate touch) and peripheral anterior synechiae (PAS) were recorded and the number of clock hours of involvement was measured. These anterior segment parameters were assessed for each meridian.

The difference in the anterior chamber parameters between primary and secondary KPros was tested using the two-tailed Student’s *t*-test for unpaired samples. All results were considered statistically significant when the p-value was less than 0.05. Microsoft Excel (Redmond, WA) was used for statistical analysis.

## Results

The series constituted 52 eyes with implanted Boston type 1 KPro. There were a total of 49 patients of whom 33 were male and the mean age at time of surgery was 56.5 years (range 26–83 years). There were 80.8% (42/52 eyes) aphakic and 19.2% (10/52 eyes) pseudophakic, and 38.5% (20/52 eyes) primary and 61.5% (32/52 eyes) secondary KPros implanted. For the primary KPros, 16 (80%) eyes had 7.0 mm and 4 (20%) eyes had 8.5 mm diameter back plates implanted. For the secondary KPros, 24 (75%) eyes had 7.0 mm and 8 (25%) eyes had 8.5 mm diameter back plates implanted. Preoperative diagnoses included graft failure (61.5%), chemical burns (13.5%), aniridia (13.5%) and Stevens-Johnson syndrome (5.8%). The mean single postoperative time point of AS-OCT imaging was 18.7 months for primary KPro, 19.6 months for secondary KPro and 19.3 months (range 0.13 to 59.13 months) for all eyes.

**Figure 1:**
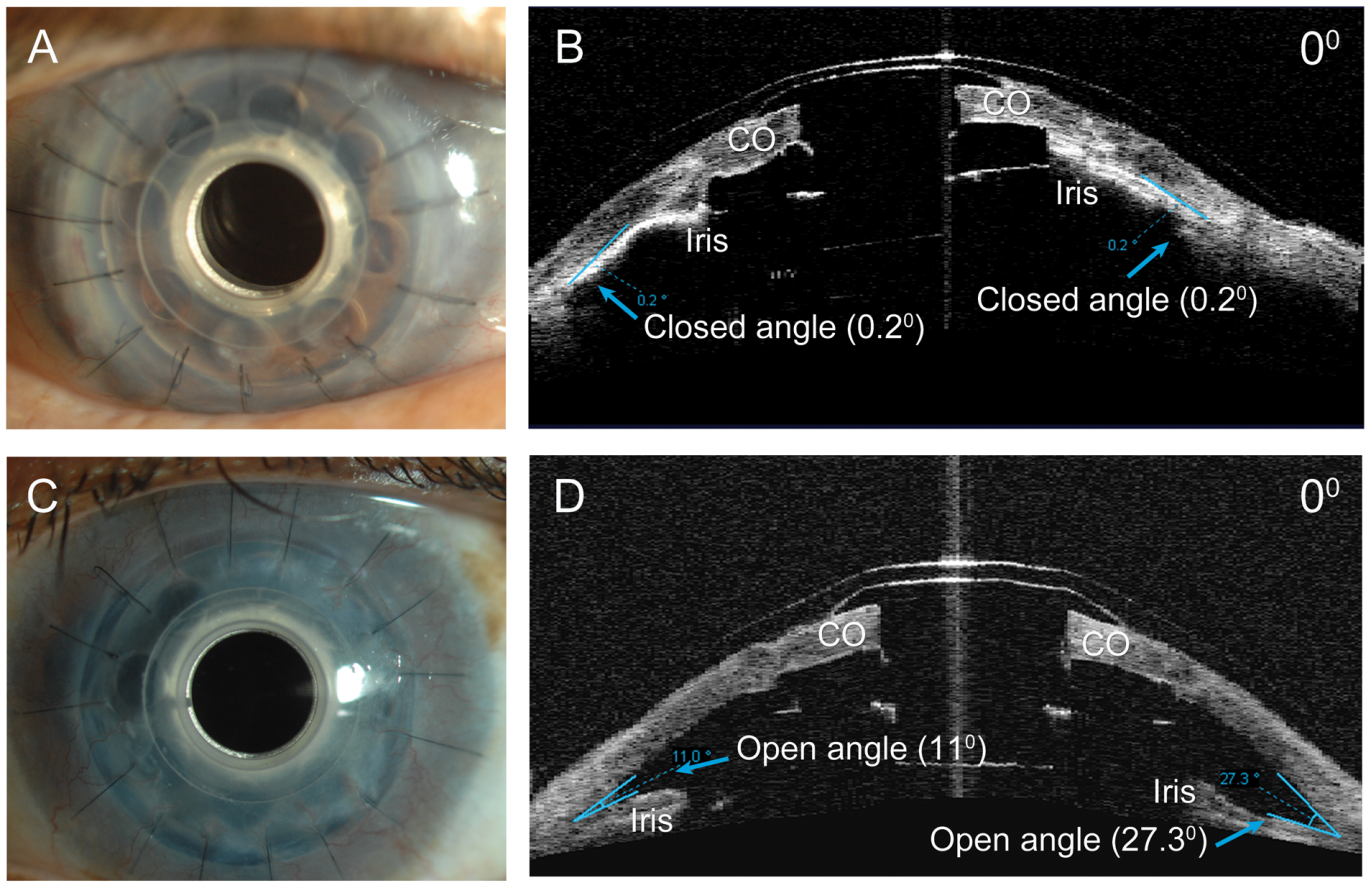
Boston keratoprosthesis and open and closed angles on AS-OCT imaging. A, Slit-lamp photograph of implanted secondary KPro for multiple graft failure. Note that details of iris behavior and angle cannot be visualized clinically. B, Horizontal AS-OCT of the same eye with irido-corneal adhesion and closed angle. C, Slit-lamp photo of another eye with implanted primary KPro for gelatinous drop like dystrophy. Note again that details of the angle cannot be visualized and appears clinically identical to eye in part A. D, Horizontal AS-OCT of eye in part C with open angle. CO = donor cornea.

AS-OCT adequately imaged the implanted KPro, anterior chamber depth and angle (Figure 1). The average anatomical ACD was 2.0 mm (range 1.49–2.57 mm) and functional ACD was 0.21 mm (range 0–1.69 mm) for all eyes. There were no significant differences in anatomical or functional ACD between primary and secondary, aphakic and pseudophakic, and the 7.0 mm and 8.5 mm back plate KPros.

The mean anterior chamber angle for all eyes was 4.76° (range 0 to 40.9°) and 4.79° (range 0 to 34.4°) at the 0° and 180° meridians, respectively. However, 73.1% (38/52 eyes) of all eyes had closed angles (<1°) at the 0° and 180° meridians. In those eyes in whom additional meridians were scanned, the mean ACA in the other measured meridians (45°, 90°, 135°, 225°, 270° and 315°) ranged from 2.5° to 6.14° (Table 1). There was a trend for secondary KPros to have decreased ACA compared with primary KPros and this difference was statistically significant in 3 measured meridians (45°, 90°, 180°). In the 25 (48.1%) eyes with all representative meridians imaged, there was an average of 8.7 clock hours of closed angle, 1.5 clock hours of shallow angle and 1.8 clock hours of open angle. This represented an average of 269 degrees of angle closure and 72.3% of the total angle was closed.

**Table 1:** Mean Anterior Chamber Angle Measurements using Anterior Segment Optical Coherence Tomography in Eyes with Boston Keratoprosthesis Type 1.

	AS-OCT Scan Direction							
	0°	45°	90°	135°	180°	225°	270°	315°
Primary KPro (n = 20)	6.72°	8.05°	11.47°	7.99°	8.89°	5.52°	4.21°	2.81°
	(n = 20)	(n = 15)	(n = 13)	(n = 15)	(n = 20)	(n = 15)	(n = 11)	(n = 14)
Secondary KPro (n = 32)	3.53°	1.65°	2.29°	2.80°	2.23°	2.74°	1.69°	2.47°
	(n = 32)	(n = 19)	(n = 18)	(n = 21)	(n = 32)	(n = 25)	(n = 23)	(n = 23)
P value (Primary vs. Secondary)	0.235	*0.015	*0.009	0.066	*0.014	0.232	0.242	0.872
All KPro (n = 52)	4.76°	4.48°	6.14°	4.96°	4.79°	3.83°	2.50°	2.61°
	(n = 52)	(n = 34)	(n = 31)	(n = 36)	(n = 52)	(n = 40)	(n = 34)	(n = 37)

KPro = keratoprosthesis; Asterisk* denotes statistical significance (p<0.05).

**Figure 2:**
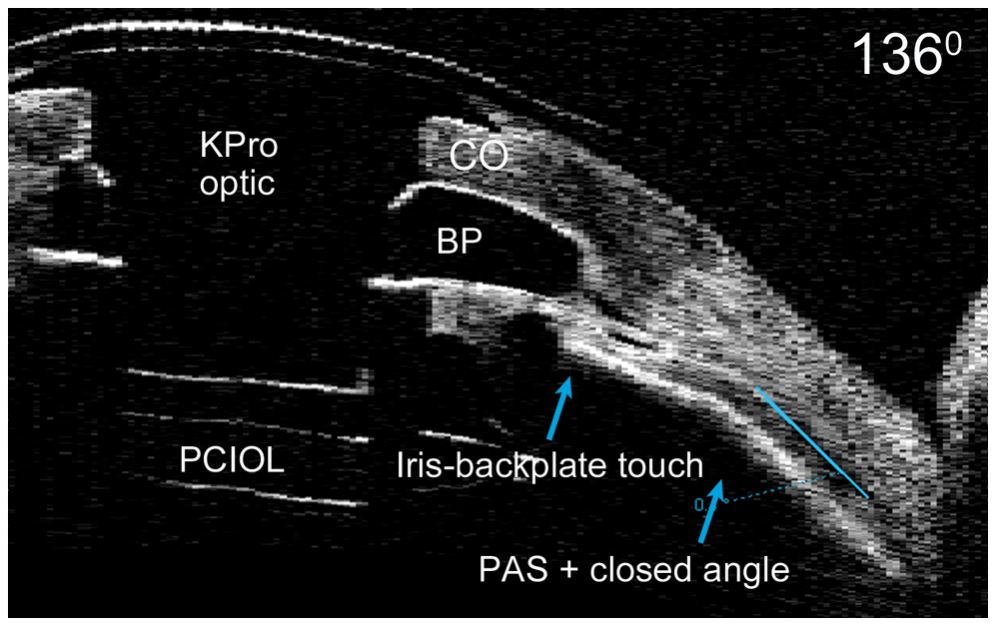
AS-OCT of implanted Boston keratoprosthesis with proximal and peripheral synechiae. Oblique AS-OCT image with iris-backplate touch, peripheral anterior synechiae and closed anterior chamber angle. CO = donor graft; PCIOL = posterior chamber intraocular lens; PAS = peripheral anterior synechiae.

Peripheral anterior synechiae was seen in forty-five (86.5%) eyes with a mean of 6.5 clock hours of iris adhesion, often developing in the same meridians of angle closure (Table 2). In addition, 35 (67.3%) eyes showed evidence of iris-back plate touch with a mean of 6.1 clock hours of proximal adhesion to the back plate (Figure 2). The rates of both peripheral and proximal synechiae development were similar for primary and secondary KPros, although there was a statistically significant greater extent (number of clock hours) of synechiae formation in secondary KPros.

**Table 2:** Peripheral and Proximal Iris Synechiae Measured by Anterior Segment Optical Coherence Tomography in Eyes with Boston Keratoprosthesis Type 1.

	Peripheral Synechiae		Iris-Backplate Touch		
	No. of eyes (%)	Mean clock hours	No. of eyes (%)	Mean clock hours	
Primary KPro (n = 20)	17 (85%)	4.9	14 (70%)	4.7	
Secondary KPro (n = 32)	28 (87.5%)	7.4	21 (65.6%)	7.0	
P value (Primary vs. Secondary)		*0.031		*0.037	
All KPro (n = 52)	45 (86.5%)	6.5	35 (67.3%)	6.1	

There were a small number of patients with aniridia (7 eyes) included in the study. For all these patients, a small stump of iris was present which allowed for analysis of the angle and evaluation of synechiae formation. When analyzed separately, there were no significant differences in ACD, ACA or synechiae formation compared to the non-aniridic group. However our sample size of aniridic patients may be too small for meaningful analysis.

## Discussion

AS-OCT offers an important imaging modality for evaluating anterior segment anatomy after KPro implantation, which has been well documented by previously published case series. [3], [11], [13] However, to our knowledge, this is the first paper to employ AS-OCT to quantitatively evaluate the anterior chamber depth, angle status and incidence of synechiae formation after KPro implantation. This is important because the KPro optic does not allow gonioscopic visualization of the anterior chamber angle.

In this current study, the majority of eyes had shallow anatomic and functional anterior chamber depths, with 82.7% of eyes exhibiting a completely flat effective (functional) anterior chamber. The majority (90.4%) of eyes showed angle closure in at least one meridian and in the 25 (48.1%) eyes with all representative meridians imaged, 73.4% (8.7 clock hours) of the angle was completely closed. Corresponding to the areas of angle closure, extensive PAS and iris-back plate touch were also observed. These findings may reflect previous synechial angle closure or may be a result of crowding of the anterior segment by the KPro back plate, which is placed in close position to the iris and in turn may compromise the angle. Because of this, we routinely use pediatric size back plates (7 mm) and prefer aphakic KPros in the majority of our patients. There was a trend toward increased ACA with the smaller size back plates, however the rates of synechiae involvement and anterior chamber depths were similar. As the present study had a disparate size of each group (13 eyes with the 8.5 mm and 39 eyes with the 7.0 mm back plate), further studies are needed to evaluate if there is a true effect of back plate size and angle closure.

Interestingly, there was a trend of decreased ACA in secondary KPro compared with primary implantation. The difference in anterior chamber angle was statistically significant in only 3 of the 8 imaged meridians; however, larger studies are needed to investigate whether this difference is significant. In addition, although the percentage of eyes with presence of peripheral and proximal synechiae were similar between both groups, the extent of involvement by synechial formation was significantly greater in secondary KPros. Previous studies have demonstrated PAS formation and secondary angle closure after PKP. [15], [16], [17], [18] It is hypothesized that this may be an important mechanism for intraocular pressure elevation and glaucoma post-PKP. We suspect that a certain extent of synechial angle closure is present in eyes with multiple graft failure and this may account for the greater degree of synechiae and angle closure in secondary KPros.

Importantly, these changes in the angle may have significant clinical implications, as angle closure may be one of the causative factors in glaucoma associated with KPro. Our results support that most patients have evidence of angle closure, however these changes may reflect pre-existing synechial angle closure. Further studies with preoperative imaging and serial postoperative imaging are needed to confirm whether these anatomical changes are induced by KPro implantation or were present previously.

In conclusion, AS-OCT is a valuable and noninvasive imaging tool that may be successfully employed to quantitatively evaluate the anterior chamber, angle status and presence of synechiae following KPro implantation. In our study, the majority of eyes were found to have shallow ACD, extensive angle closure and synechiae formation. AS-OCT offers an important clinical perspective in understanding anterior segment dynamics and may be used as a tool to monitor angle closure following KPro implantation.

## References

[1] Dohlman CH, Harissi-Dagher M, Khan BF, Sippel K, Aquavella JV, et al (2006) Introduction to the use of the Boston keratoprosthesis. Expert Rev Ophthalmol 1: 41–48.

[2] Khan BF, Harissi-Dagher M, Khan DM, Dohlman CH (2007) Advances in Boston keratoprosthesis: enhancing retention and prevention of infection and inflammation. International ophthalmology clinics 47: 61–71. 17450007 10.1097/IIO.0b013e318036bd8b

[3] Garcia JP Jr, de la Cruz J, Rosen RB, Buxton DF (2008) Imaging implanted keratoprostheses with anterior-segment optical coherence tomography and ultrasound biomicroscopy. Cornea 27: 180–188. 18216573 10.1097/ICO.0b013e318159bc7d

[4] Garcia JP Jr, Garcia PM, Buxton DE, Panarelli A, Rosen RB (2007) Imaging through opaque corneas using anterior segment optical coherence tomography. Ophthalmic surgery, lasers & imaging : the official journal of the International Society for Imaging in the Eye 38: 314–318. 10.3928/15428877-20070701-07 17674922

[5] Pavlin CJ, Harasiewicz K, Sherar MD, Foster FS (1991) Clinical use of ultrasound biomicroscopy. Ophthalmology 98: 287–295. 2023747 10.1016/s0161-6420(91)32298-x

[6] Abbasian J, Cortina MS, de la Cruz J (2011) Use of preoperative imaging for surgical planning in patients undergoing Boston keratoprosthesis type 1. Techniques in Ophthalmology 9: 71–73.

[7] Bell NP, Feldman RM, Zou Y, Prager TC (2008) New technology for examining the anterior segment by ultrasonic biomicroscopy. Journal of cataract and refractive surgery 34: 121–125. 18165091 10.1016/j.jcrs.2007.09.016

[8] Radhakrishnan S, Rollins AM, Roth JE, Yazdanfar S, Westphal V, et al (2001) Real-time optical coherence tomography of the anterior segment at 1310 nm. Archives of ophthalmology 119: 1179–1185. 11483086 10.1001/archopht.119.8.1179

[9] Fernandez AG, Radcliffe NM, Sippel KC, Rosenblatt MI, Sood P, et al (2012) Boston type I keratoprosthesis-donor cornea interface evaluated by high-definition spectral-domain anterior segment optical coherence tomography. Clinical ophthalmology 6: 1355–1359. 22969280 10.2147/OPTH.S34787 PMC3437949

[10] Kiang L, Rosenblatt MI, Sartaj R, Fernandez AG, Kiss S, et al (2012) Surface epithelialization of the type I Boston keratoprosthesis front plate: immunohistochemical and high-definition optical coherence tomography characterization. Graefe’s archive for clinical and experimental ophthalmology = Albrecht von Graefes Archiv fur klinische und experimentelle Ophthalmologie 250: 1195–1199. 10.1007/s00417-012-1960-5 PMC3404271 22371021

[11] Garcia JP Jr, Ritterband DC, Buxton DF, De la Cruz J (2010) Evaluation of the stability of Boston type I keratoprosthesis-donor cornea interface using anterior segment optical coherence tomography. Cornea 29: 1031–1035. 20517152 10.1097/ICO.0b013e3181ca2ea5

[12] Basu S, Taneja M, Sangwan VS (2011) Boston type 1 keratoprosthesis for severe blinding vernal keratoconjunctivitis and Mooren’s ulcer. International ophthalmology 31: 219–222. 21424885 10.1007/s10792-011-9438-8

[13] Panarelli JF, Ko A, Sidoti PA, Garcia JP, Banitt MR (2012) Angle Closure After Boston Keratoprosthesis. Journal of glaucoma 2012 May 16. [Epub ahead of print]. 10.1097/IJG.0b013e318259b2fc 22595935

[14] Jancevski M, Foster CS (2010) Anterior segment optical coherence tomography. Seminars in ophthalmology 25: 317–323. 21091018 10.3109/08820538.2010.518473

[15] Dada T, Aggarwal A, Vanathi M, Gadia R, Panda A, et al (2008) Ultrasound biomicroscopy in opaque grafts with post-penetrating keratoplasty glaucoma. Cornea 27: 402–405. 18434841 10.1097/ICO.0b013e31816373c5

[16] Gilvarry AM, Kirkness CM, Steele AD, Rice NS, Ficker LA (1989) The management of post-keratoplasty glaucoma by trabeculectomy. Eye 3 (Pt 6): 713–718. 10.1038/eye.1989.110 2630351

[17] Cohen EJ, Kenyon KR, Dohlman CH (1982) Iridoplasty for prevention of post-keratoplasty angle closure and glaucoma. Ophthalmic surgery 13: 994–996. 6761618

[18] Lass JH, Pavan-Langston D (1979) Timolol therapy in secondary angle-closure glaucoma post penetrating keratoplasty. Ophthalmology 86: 51–59. 394061 10.1016/s0161-6420(79)35545-2

