# Correction: Anterior Segment Optical Coherence Tomography for the Quantitative Evaluation of the Anterior Segment Following Boston Keratoprosthesis

**DOI:** 10.1371/annotation/c3ad2f44-5b6f-430a-b5cb-993eae6644ad

**Published:** 2013-09-24

**Authors:** Joann J. Kang, Norma Allemann, Thasarat S. Vajaranant, Jose de la Cruz, Maria Soledad Cortina

The name of the third author was given incorrectly. The correct name is: Thasarat S. Vajaranant. 

The correct citation is: Kang JJ, Allemann N, Vajaranant TS, de la Cruz J, Cortina MS (2013) Anterior Segment Optical Coherence Tomography for the Quantitative Evaluation of the Anterior Segment Following Boston Keratoprosthesis. PLoS ONE 8(8): e70673. doi:10.1371/journal.pone.0070673. 

The correct information in the Author Contributions statement is: Conceived and designed the experiments: JJK NA TSV JDLC MSC. Performed the experiments: JJK NA TSV JDLC MSC. Analyzed the data: JJK NA TSV JDLC MSC. Wrote the paper: JJK NA TSV JDLC MSC. 

